# Supplementary material for: Understanding the connection between hospital goals and patient and family engagement: A scoping review
Source: PLoS One. 2023 Oct 26;18(10):e0293013. doi: 10.1371/journal.pone.0293013 (PMC10602333; doi:10.1371/journal.pone.0293013)
Supplement: S1 File — (DOCX) [file pone.0293013.s001.docx]

**Understanding the Connection between Hospital Goals and Patient and Family Engagement: A Scoping Review**

Supplementary File

[**Search Strategy (MEDLINE)** 2](#_Toc124752065)

[**Grey Literature Search Sources** 3](#_Toc124752066)

[**Eligibility Criteria – Title and Abstract Screening** 4](#_Toc124752067)

[**Eligibility Criteria – Full-text Screening** 5](#_Toc124752068)

[**Study Characteristics** 7](#_Toc124752069)

[**Methodological Characteristics** 11](#_Toc124752070)

[**Detailed Analysis of Study and Methodological Characteristics** 14](#_Toc124752071)

# **Search Strategy (MEDLINE)**

**Patient Engagement Filter**

1/ exp patient-centered care/ {22834}

2/ ((patient* OR famil* OR caregiver* OR care partner* OR consumer* OR user* OR client*) adj2 engag*).tw,kw {14017}

3/ ((patient* OR famil* OR caregiver* OR care partner* OR consumer* OR user* OR client*) adj2 involv*).tw,kw {59942}

4/ ((patient* OR famil* OR caregiver* OR care partner* OR consumer* OR user* OR client*) adj2 participat*).tw,kw {30778}

5/ patient participation/ {28225}

6/ exp Patient Satisfaction/ {96668}

7/ patient value*.tw,kw {1134}

8/ Patient Reported Outcome Measures/ {10771}

9/ Patient Outcome Assessment/ {5554}

10/ or/1-9 {247559}

**Hospital Strategic Planning Filter**

11/ Hospital Planning/og [Organization & Administration] {656}

12/ Decision Making/ {101539}

13/ Organizational Objectives/ {21097}

14/ Health Services Administration/og [Organization & Administration] {59}

15/ Quality Improvement/og [Organization & Administration] {4817}

16/ Hospital Administration/og [Organization & Administration] {460}

17/ Health Facility Administration/ {1951}

18/ Quality of Health Care/og [Organization & Administration] {4507}

19/ ((organi$ation* OR institution* OR hospital*) adj2 (mission* OR vision* OR value*)).tw,kw {2635}

20/ ((mission* OR vision* OR value*) adj3 statement*).tw,kw {1076}

21/ leadership/ {44942}

22/ Health Facilities/ {17159}

23/ or/11-22 {194264}

**Country Filter**

24/ Canada/ {101190}

25/ United States/ {945167}

26/ United Kingdom/ {2407110}

27/ Australia/ {116257}

28/ New Zealand/ {42450}

29/ Europe/ {1486297}

30/ or/24-29 {1486297}

31/ 10 and 23 and 30 {3123}

# **Grey Literature Search Sources**

| Sources | Search Terms |
| --- | --- |
| Corporate Websites   - [Patient-Centered Outcomes Research Institute](https://www.pcori.org/explore-our-portfolio?keyword=) - [North American Observatory on Health Systems and Policies](https://ihpme.utoronto.ca/tag/nao/) - [Institute of Health Policy, Management and Evaluation](https://ihpme.utoronto.ca/research/policy-papers/) - [Institute for Patient and Family Centered Care](https://www.ipfcc.org/)   Journals   - [Patient Experience Journal](https://pxjournal.org/journal/) - [Journal of Patient Experience](https://journals.sagepub.com/home/jpx) - [Health Expectations](https://onlinelibrary.wiley.com/journal/13697625) - [Health Affairs](https://www.healthaffairs.org/) - [BMC Research Involvement and Engagement](https://researchinvolvement.biomedcentral.com/) - [Healthcare Management Forum](https://journals.sagepub.com/home/hmf) - [Milbank Quarterly](https://onlinelibrary.wiley.com/journal/14680009)   Other   - [Open Grey](https://opengrey.eu/) | 1. Mission  2. Vision  3. Value  4. Patient  5. Engagement  6. Involvement  7. Participation |

# **Eligibility Criteria – Title and Abstract Screening**

| **Population** | - Patients, family members, informal caregivers, and patient and family partners 18 years of age and above with experience of any disease, medical condition, or health care issue requiring a hospital visit/stay. - Clinicians, including physicians, nurses, or other health care providers. - Executives and managers involved in health service planning and improvement activities or the development and execution of MVV statements. - English-language publications on any healthcare setting. - Articles published between 2012 and 2022. |
| --- | --- |
| **Intervention or Issue** | - Studies that specifically discuss the following: 1) MVV statements (or other ways to represent and communicate organizational goals), and 2) the connection or impact of these statements on PE processes and activities in health service organizations. - Included studies do not need to focus on MVV statements (or other representations of goals) as the study's primary objective. Still, they need to mention it explicitly in the title, abstract, findings, or discussion sections. - Health service organizations might represent or communicate their goals in the following ways: strategic plans, strategies, aspirations, mission statements, vision statements, policy statements, patient declaration of values, patient bill of rights and responsibilities, etc. |
| **Outcomes** | - Process outcomes from eligible studies may include strategies or tools used to promote MVV statements; how these statements translate into PE processes and activities; determinants that clarify the connection between MVV statements, and PE processes and activities; staff or patient, or family perceptions of MVV statements and/or the strategies and tools used to promote them; and other process outcomes on participants, facilities, services, and patient care |
| **Comparison / Study Designs** | N/A |
| **Exclusions** | - Studies that do not explore or discuss the connection between MVV (or other representations of goals) statements, and PE processes and activities - Studies conducted in settings outside of health service organizations. The study settings will be kept broad to ensure that the maximum number of relevant studies are retrieved - Studies focused on treatment goals or shared decision-making, or any studies not directly tied to goals or values (or related concepts) unless patient-centred care or patient engagement or experience language is used - Studies that discuss patient engagement (or related concepts) or goals (or related concepts) but not both. |

# **Eligibility Criteria – Full-text Screening**

| **Population** | - Patients, family members, informal caregivers, and patient and family partners 18 years of age and above with experience of any disease, medical condition, or health care issue requiring a hospital visit/stay. - Clinicians, including physicians, nurses, or other health care providers. - Executives and managers involved in health service planning and improvement activities or the development and execution of MVV statements. - English-language publications on any healthcare setting are published in Canada, the United States, Australia, and the European Economic Area since empirical research on PE generally originates from countries with similar economic and health systems. - Articles published between 2012 and 2022. |
| --- | --- |
| **Intervention or Issue** | - Studies that specifically discuss the following: 1) MVV statements (or other ways to represent and communicate organizational goals), and 2) the connection or impact of these statements on PE processes and activities in health service organizations. - Included studies do not need to focus on MVV statements (or other representations of goals) as the study's primary objective. Still, they need to mention it explicitly in the title, abstract, findings, or discussion sections. - Health service organizations might represent or communicate their goals in the following ways: strategic plans, strategies, aspirations, mission statements, vision statements, policy statements, patient declaration of values, patient bill of rights and responsibilities, etc. Unit of analysis: any health service organization including but not limited to the following: hospitals, clinics, health care agencies (e.g., health technology assessment agencies), health institutions, organizational models (e.g., patient-centered medical home), and professional associations |
| **Outcomes** | - Process outcomes from eligible studies may include strategies or tools used to promote MVV statements; how these statements translate into PE processes and activities; determinants that clarify the connection between MVV statements, and PE processes and activities; staff or patient or family perceptions of MVV statements and/or the strategies and tools used to promote them; and other process outcomes on participants, facilities, services, and patient care |
| **Comparison / Study Designs** | N/A |
| **Exclusions** | - Studies that do not explore or discuss the connection between MVV (or other representations of goals) statements, and PE processes and activities - Studies conducted in settings outside of health service organizations. The study settings will be kept broad to ensure that the maximum number of relevant studies are retrieved - Studies focused on treatment goals or shared decision-making, or any studies not directly tied to goals or values (or related concepts) unless patient-centred care or patient engagement or experience language is used - Studies that discuss patient engagement (or related concepts) or goals (or related concepts) but not both. |

# **Study Characteristics**

| **Author, Year**  **Country** | **Title** | **Objectives** | **Study Design – Methodology or Analytic Method** | **Data Collection Method(s)** |
| --- | --- | --- | --- | --- |
| Beckett, 2013  Australia | Practice what you preach: developing person-centred culture inpatient mental health settings through strengths-based, transformational leadership | Not reported | Qualitative – not specified | Not reported |
| Berman, 2016  United States | Why human resources policies and practices are critical to improving the patient experience | Argue that human resource policies, procedures and programs are key to supporting an organization's vision and culture | Commentary | Not applicable |
| Bokhour, 2018  United States | How can healthcare organizations implement patient-centred care? Examining a large-scale cultural transformation | Understand key organizational factors for implementing patient-centered care cultural transformation through an examination of efforts in the US Department of Veterans Affairs | Qualitative – constant comparison | Semi-structured interviews |
| Bridge, 2016  Canada | Using patient value statements to develop a culture of patient-centred care: a case study of an Ontario, Canada hospital | Explore how a leading patient-centred Ontario hospital operationalized their patient value statement in policy and practice | Qualitative – case study | Semi-structured interviews |
| Bromley, 2012  United States | Building patient-centeredness: hospital design as an interpretive act | Describe the impact of the concept of patient-centeredness on the design of a new hospital in the United States | Qualitative – not reported | Semi-structured interviews |
| D'Agostino, 2017  United States | Patient experience in the behavioral health setting: key best practices throughout an organizational journey | Describe the organizational journey that led to these improvements and identify learning for other hospitals, particularly in a behavioural healthcare setting | Commentary | Not applicable |
| Dickson, 2018  United Kingdom | "It's a nice place, a nice place to be": the story of a practice development program to further develop person-centred cultures in palliative and end-of-life care | Implement a program of practice development to further the development of a culture of person-centred practice in the Marie Curie Care Edinburge Hospice | Qualitative – participatory action research | Stories, observation |
| Dokken, 2020  United States | Families as care partners: implementing the Better Together initiative across a large health system | Describe the growth of Better Together: Partnering with Families, a campaign launched in 2014 to eliminate restrictive hospital visiting policies and to put in place policies that recognize families as partners in care | Descriptive report | Not applicable |
| Frakking, 2020  Australia | Framework for patient, family-centred care within an Australian community hospital: development and description | Describe the development of a patient and family-centred care conceptual framework within a small community Australian hospital | Scoping review | Not applicable |
| Gilligan, 2019  United States | Views of institutional leaders on maintaining humanism in today's practice | Explore leadership perspectives on how to maintain high quality efficient care that is also person-centered and humanistic | Qualitative – not reported | Semi-structured interviews |
| Hatlie, 2020  United States | Lessons learned from a systems approach to engaging patients and families in patient safety transformation | Provide lessons learned six years after establishing an infrastructure of patient and family advisory councils focused on health care quality and safety | Descriptive report | Not applicable |
| Hayward, 2014  United States | A focus on 'Always Events': strategy ensures patient-centered care and a better patient experience | Not reported | Descriptive report | Not applicable |
| Hernandez, 2013  United States | Patient-centered innovation in health care organizations: a conceptual framework and case study application | Propose a framework for understanding the process of initiating patient-centered innovations | Narrative review | Not reported |
| Kang, 2013  United States | Hospital commitment to community orientation and its association with quality of care and patient experience | Examine the association between hospital community orientation and quality-of-care measures | Quantitative – retrospective review | 2009 American Hospital Association's Annual Survey Database |
| Kokorelias, 2021  Canada | Assessing readiness to implement patient navigator programs in Toronto, Canada | Explore factors influencing the implementation of patient navigator programs within a hospital for seniors with complex care needs | Qualitative – thematic analysis | Semi-structured interviews |
| Manalili, 2022  Canada | Informing the implementation and use of person-centered quality indicators: a mixed methods study on the readiness, barriers and facilitators to implementation in Canada | Assess the readiness of Canadian healthcare organizations and explored their percieved barriers and facilitators to implementing and using person-centered quality indicators | Mixed methods | Survey and semi-structured interviews |
| Mahas, 2020  Canada | Exploring patient centredness, communication and shared decision-making under a new model of care: community rehabilitation in Canada | Describe how patients and providers experienced SDM at community rehabilitation sites that adopted a novel, patient-centered rehabilitation model of care | Qualitative – ethnography | Focus groups and semi-structured interviews |
| Metusela, 2020  Australia | Patient Centred Medical Home (PCMH) transitions in western Sydney, Australia: a qualitative study | Explore the experiences of healthcare providers in outer urban Sydney, where a number of practices are transitioning from traditional Australian general practice models to incorporate elements of PCMH approaches | Qualitative - thematic analysis | Semi-structured interviews |
| O’Connor, 2018  Canada | The leadership and organizational context required to support patient partnerships | Describe challenges in creating a culture of patient partnerships and the leadership actions and organizational context required now and in the future to support engagement-capable environments at the organizational and policy levels in Canada | Descriptive report | Not applicable |
| Pandhi, 2020  United States | Engaging patients in primary care quality improvement initiatives: facilitators and barriers | Determine the most effective strategies for disseminating a previously successful single-system patient engagement in quality improvement intervention | Qualitative – not reported | Semi-structured interviews, observations, visits, and journaling |
| Pruthi, 2015  United States | Vision, mission, and values: from concept to execution at Mayo Clinic | Share the Mayo Model of Care and patient stories that embody the 8 Mayo Clinic values of respect, compassion, integrity, healing, teamwork, excellence, innovation, and stewardship | Descriptive report | Not applicable |
| Reed, 2012  United States | Innovation in patient-centered care: lessons from a qualitative study of innovative health care organizations in Washington State | Understand the process of patient-centered care innovation undertaken by innovative health care organizations - from strategic planning to goal selection to implementation to maintenance | Qualitative – not reported | Semi-structured interviews |
| Scholtes, 2020  Multiple - Belgium, France, Germany, and Luxembourg | Hospital practices for the implementation of patient partnership in a multi-national European region | Assess the occurrence of patient participation and involvement practices in hospitals in Belgium, France, Germany and Luxembourg, and analyze if, and to what extent, the hospital vision and the presence of patient committee influence the implementation of PPI practices | Quantitative – cross-sectional | Survey |
| Skingley, 2021  United Kingdom | Implementing the PIE (Person, Interaction, and Environment) program to improve person-centred care for people with dementia admitted to hospital wards: a qualitative evaluation | Describe and evaluate PIE implementation in three UK NHS regions | Qualitative – case study | Semi-structured interviews, observations, document analysis |
| Taylor, 2015  Europe – not specified | European hospital manager's perceptions of patient-centred care: a qualitative study on implementation and context | Assess the perceptions of European hospital management with respect to factors affecting the implementation of a patient-centred approach | Qualitative - framework analysis | Semi-structured interviews |
| Venturato, 2011  Australia | Exploring the gap between rhetoric and reality in dementia care in Australia: could practice documents help bridge the great divide? | Identify key documents guiding dementia care within one large Australian long-term care organization, explore points of consistency and tension within the documented system of care | Qualitative – ethnography | Document analysis |
| Wright, 2014  United States | Mission, margin, and the role of consumer governance in decision-making at community health centers | Explore the role of consumer trustees in decision-making as community health centers work to navigate the tension between pursuing their mission to provide primary care to all regardless of ability to pay and maintaining their limited finances | Qualitative – not reported | Semi-structured interviews |

# **Methodological Characteristics**

| **Author, Year** | **Sample Size** | **Topic Context (detail)** | **Health Care Context** |
| --- | --- | --- | --- |
| Beckett, 2013 | Not reported | Specific intervention (Away Days) | Mental health/ psychiatric hospital |
| Berman, 2016 | Not applicable | Organizational implementation of patient-centered care (role of HR) | General/not specified |
| Bokhour, 2018 | 108 employees (22 senior leaders, 42 middle managers, 37 front-line providers, and 7 staff) | Organizational implementation of patient-centered care | Veteran Affairs |
| Bridge, 2016 | 18 (1 CEO, 1 PCC lead, 4 directors and clinical managers of programs, 1 physician, 1 nurse practitioner, 2 nurses, 2 allied health professionals, a maintenance worker, a porter, and 2 patient/family advisors) | Relationship between philosophies / frameworks and practices (patient declaration of values) | General/not specified |
| Bromley, 2012 | 35 planners, administrators, and designers of new hospital | Organizational implementation of patient-centered care | General/not specified (new hospital) |
| D'Agostino, 2017 | Not applicable | Organizational implementation of patient-centered care (post-merger) | Mental health/ psychiatric hospital |
| Dickson, 2018 | Not reported | Specific intervention (patient-centered care practice development program) | Palliative care |
| Dokken, 2020 | Not applicable | Specific intervention (Better Together) | General/not specified |
| Frakking, 2020 | 107 publications | Relationship between philosophies / frameworks and practices (Patient and Family Centered Care conceptual framework) | Community hospital |
| Gilligan, 2019 | 32 institutional leaders at 7 medical schools | Organizational implementation of patient-centered care (humanism in health care) | General/not specified (academic hospital) |
| Hatlie, 2020 | Not applicable | Specific intervention (Patient and Family Advisory Council) | General/not specified |
| Hayward, 2014 | Not applicable | Specific intervention (Always Events) | General/not specified |
| Hernandez, 2013 | Not applicable | Organizational implementation of patient-centered care (patient-centered care innovation) | General/not specified (innovation health care organization) |
| Kang, 2013 | 3556 hospitals | Relationship between philosophies / frameworks and practices (hospital community orientation) | General/not specified |
| Kokorelias, 2021 | 13 non-clinical managers or administrators, 22 clinicians (social workers, OTs, PTs, nurses, and physicians) | Organizational implementation (readiness for patient navigator programs) | General/not specified |
| Manalili, 2022 | 33 organizations responded to survey, interviews with 42 participants | Organizational implementation (readiness to use person-centered care indicators for quality improvement) | General/not specified |
| Manhas, 2020 | 45 providers and 17 patients | Organizational implementation (rehabilitation model of care) | Community hospital or centre |
| Metusela, 2020 | 4 primary care physicians, 28 managers, 3 nurses | Organizational implementation of patient-centered care (transition to PCMH) | Primary care |
| O’Connor, 2018 | Not applicable | Organizational implementation of patient-centered care (patient partnerships) | General/not specified |
| Pandhi, 2020 | 8 primary care clinics | Organizational implementation of patient-centered care (Patient engagement and quality improvement innovations) | Primary care |
| Pruthi, 2015 | Not applicable | Relationship between philosophies / frameworks and practices | General/not specified |
| Reed, 2012 | 5 health plans (health insurance providers), 5 provider organizations, and 10 clinics | Organizational implementation of patient-centered care (patient-centered care innovation) | General/not specified (innovative health care organization) |
| Scholtes, 2020 | 64 hospitals, 54% were hospital managers | Specific intervention (patient and public involvement) | General/not specified |
| Skingley, 2021 | 10 wards | Specific intervention (person, interaction, and environment program) | Dementia care |
| Taylor, 2015 | 10 hospital managers, 2 expert country informants, 2 patient organizations, and 1 user representative | Organizational implementation of patient-centered care | General/not specified |
| Venturato, 2011 | Documents across 25 long-term care facilities | Relationship between philosophies / frameworks and practices (documented systems of care) | Dementia Care |
| Wright, 2014 | 30 trustees from 16 community health centers across 14 states | Relationship between philosophies / frameworks and practices | Community hospital |

# **Detailed Analysis of Study Characteristics**

Country and Year of Publication

All articles were published after 2011, with 12 (44.4%) published between 2011-2016 and 15 (55.6%) between 2017-2022. Most studies were published in the United States (n = 14, 51.9%) [1-14]. Studies were also published in Canada (n = 5, 18.5%) [15-19], 2018 #45}, Australia (n = 4, 14.8%) [20-23], the United Kingdom (n = 2, 7.4%) [24, 25], and multiple countries (n = 2, 7.4%) [26, 27].

Study Design

Studies adopted primarily a qualitative study design (n = 15, 55.6%) [2, 3, 6, 11, 13-16, 18, 20, 22-25, 27]. Six of these studies did not specify the qualitative study design [3, 6, 11, 13, 14, 20]. Two studies adopted a case study approach [Bridge 2016, Skingley 2021] and ethnography [18, 23], and one study adopted a participatory action research approach [24]. As for the analytic approach, two studies employed thematic analysis [16, 22], framework analysis [27], and constant comparison [2].

Other types of studies included descriptive reports (n = 5, 18.5%) [5, 7, 8, 12, 19], quantitative study designs (n = 2, 7.4%) [10, 26], commentaries (n = 2, 7.4%) [1, 4], reviews (n = 2, 7.4%) [9, 21], and mixed methods study (n = 1, 3.7%) [17].

Participants

Articles included a total of 443 participants, which consisted of 207 (46.7%) administrators, directors, and managers across eight studies [2, 3, 15-17, 22, 26, 27]; 55 (12.4%) leaders across three studies [2, 6, 15]; 54 (12.2%) patient, family or community advisors across four studies [14, 15, 18, 27]; and 82 (18.5%) clinicians across five studies [2, 15, 16, 18, 22]. Two studies focused on hospital publications and documents [21, 23], and three focused on health service organizations, hospital wards, and health plans [11, 13, 25].

Setting

More articles named a specific hospital, hospital system, program, or initiative (n = 16, 59.3%) [3-5, 7-9, 12, 15, 16, 18-21, 23-25] than articles that described multiple hospitals or hospital systems, or discussed hospitals generally (n = 11, 40.7%) [1, 2, 6, 10, 13, 14, 17, 22, 26-28].

More studies did not identify a medical discipline (n = 14, 51.8%). However, four of these studies identified the setting context, such as innovative health service organizations (n = 2, 7.4%) [9, 13], academic hospitals (n = 1, 3.7%) [6], and new hospitals (n = 1, 3.7%) [3]. Of the remaining studies that identified a medical discipline included community care (n = 3, 11.1%) [14, 18, 21], mental health or psychiatric hospital (n = 2, 7.4%) [4, 20], primary care (n = 2, 7.4%) [11, 22], dementia care (n = 2, 7.4%) [23, 25], palliative care (n = 1, 3.7%) [24], and veteran care (n = 1, 3.7%) [2].

1. Berman S. Why human resources policies and practices are critical to improving the patietn experience. Patient Exp J. 2016;3(2):9-11.

2. Bokhour BG, Fix GM, Mueller NM, Barker AM, Lavela SL, Hill JN, et al. How can healthcare organizations implement patient-centred care? Examining a large-scale cultural transformation. BMC Health Serv Res. 2018;18(1):1-.

3. Bromley E. Building patient-centeredness: hospital design as an interpretive act. Soc Sci Med. 2012;75(6):1057-66.

4. D'Agostino ML, Vizner T, Wald D, Espinosa L, Evans R. Patient experience in the behavioral health setting: key best practices throughout an organizational journey. Patient Exp J. 2017;4(3):147-52.

5. Dokken D, Barden A, Tuomey M, Giammarinaro N, Johnson B. Families as care partners: implementing the Better Together initiative across a large health system. J Clin Outcomes Manag. 2020;27(1).

6. Gilligan MC, Osterberg LG, Rider EA, Derse AR, Weil AB, Litzelman DK, et al. Views of institutional leaders on maintaining humanism in today's practice. Patient Educ Couns. 2019;102(10):1911-6.

7. Hatlie MJ, Nahum A, Leonard R, Jones L, Nahum V, Krevat SA, et al. Lessons learned from a systems approach to engaging patients and families in patient safety transformation. Jt Comm J Qual Patient Saf. 2020;46(3):158-66.

8. Hayward M, Endo JA, Rutherford P. A focus on 'Always Events': strategy ensures patient-centered care and a better patient experience. Healthc Exec. 2014;29(1):78-81.

9. Hernandez SE, Conrad DA, Marcus-Smith MS, Reed P, Watts C. Patient-centered innovation in health care organizations: a conceptual framework and case study application. Health Care Manage Rev. 2013;38(2):166-75.

10. Kang R. Hospital commitment to community orientation and its association with quality of care and patient experience. J Health Manage. 2013;58(4):277-88.

11. Pandhi N, Jacobson N, Crowder M, Quanbeck A, Hass M, Davis S. Engaging Patients in Primary Care Quality Improvement Initiatives: Facilitators and Barriers. Am J Med Qual. 2020;35(1):52-62.

12. Pruthi S, Davis DM, Hucke DL, Ripple FB, Tatzel BS, Dilling JA, et al. Vision, mission, and values: from concept to execution at Mayo Clinic. Patient Exp J. 2015;2(2):168-73.

13. Reed P, Conrad DA, Hernandez SE, Watts C, Marcus-Smith M. Innovation in patient-centered care: lessons from a qualitative study of innovative health care organizations in Washington State. BMC Fam Pract. 2012;13(1):1-9.

14. Wright B, Martin GP. Mission, margin, and the role of consumer governance in decision-making at community health centers. J Health Care Poor Underserved. 2014;25(2):930.

15. Bridge E, Law MP, Narushima M. Using patient value statements to develop a culture of patient-centred care: a case study of an Ontario, Canada hospital. Patient Exp J. 2016;3(2):87-98.

16. Kokorelias KM, Gould S, DasGupta, T. C, D.,, Hitzig SL. Assessing readiness to implement patient navigator programs in Toronto, Canada. Journal of Evaluation in Clinical Practice. 2022;28(4):550-7.

17. Manalili K, Scott CM, O'Beirne M, Hemmelgarn BR, Santana MJ. Informing the implementation and use of person-centred quality indicators: a mixed methods study on the readiness, barriers and facilitators to implementation in Canada. BMJ. 2022;12(8):e060441.

18. Manhas KP, Olson K, Churchill K, Miller J, Teare S, Vohra S, et al. Exploring patient centredness, communication and shared decision‐making under a new model of care: Community rehabilitation in Canada. Health & Social Care in the Community. 2022;30(3):1051-63.

19. O'Connor P, Di Carlo M, Rouleau JL. The leadership and organizational context required to support patient partnerships. Healthc Quart. 2018;21:31-7.

20. Beckett P, Field J, Molloy L, Yu N, Holmes D, Pile E. Practice what you preach: developing person-centred culture inpatient mental health settings through strengths-based, transformational leadership. Issues Men Health Nurs. 2013;34(8):595-601.

21. Frakking T, Michaels S, rbell-Smith J, Le Ray L. Framework for patient, family-centred care within an Australian community hospital: development and description. BMJ Open Qual. 2020;9(2):e000823.

22. Metusela C, Usherwood T, Lawson K, Angus L, Kmet W, Ferdousi S, et al. Patient Centred Medical Home (PCMH) transitions in western Sydney, Australia: a qualitative study. BMC Health Serv Res. 2020;20(1):1-3.

23. Venturato L, Moyle W, Steel A. Exploring the gap between rhetoric and reality in dementia care in Australia: could practice documents help bridge the great divide? Dementia. 2013;12(2):251-67.

24. Dickson C, Smith T, Ford H, Ludwig S, Moyes R, Lee L, et al. "It's a nice place, a nice place to be": the story of a practice development programme to further develop person-centred cultures in palliative and end-of-life care. Int Prac Develop J. 2018.

25. Skingley A, Godfrey M, Henderson R, Manley K, Shannon R, Young J. Implementing the PIE (Person, Interaction, and Environment) programme to improve person-centred care for people with dementia admitted to hospital wards: a qualitative evaluation. Int Pract Develop J. 2021;11(1).

26. Scholtes B, Breinbauer M, Rinnenburger M, Voyen M, Nguyen-Thi PL, Ziegler O, et al. Hospital practices for the implementation of patient partnership in a multi-national European region. Eur J Public Health. 2020;31(1):73-9.

27. Taylor A, Groene O. European hospital manager's perceptions of patient-centred care: a qualitative study on implementation and context. J Health Org Manag. 2015;29(6):711-28.

28. Pandhi N, Jacobson N, Crowder M, Quanbeck A, Hass M, Davis S. Engaging patients in primary care quality improvement initiatives: facilitators and barriers. J Med Qual. 2020;35(1):52-62.
