# Supplementary material for: Understanding the connection between hospital goals and patient and family engagement: A scoping review
Source: PLoS One. 2023 Oct 26;18(10):e0293013. doi: 10.1371/journal.pone.0293013 (PMC10602333; doi:10.1371/journal.pone.0293013)
Supplement: Attachment — Submitted filename: MVV Rebuttal Letter 07 Sep 23.docx [file pone.0293013.s003.docx]

**Reviewer #1: This is a very well written and sophisticated scoping review of an important topic in healthcare today. It demonstrates an excellent understanding of the healthcare organisational problem, scoping review methodology and the use of theoretical and analytical frameworks to aid the review process. The links to additional research in the discussion is particularly noteworthy.**

Thank you for your positive comments. We are excited that you found this an interesting and important study.

**Just some very minor revisions needed.

First sentence of research results is a bit ambiguous. Can this be rephrased please?**

We have revised the initial sentence for clarity. It now reads as “The initial and updated searches combined yielded a total of 8992 abstracts after removing duplicates” (p. 4)

**The paragraph after Table 2 requires some clarity of writing. “This section” (do the authors mean this scoping review or the original realization factors?). I needed to read this paragraph a couple of times to fully understand the authors intent. Perhaps a sub-heading for this section: Aligning the SR with the original realization factors framework etc..would be helpful.**

Thank you for this comment. We agree that this section needed clarity. We have clarified the writing and hope our intention is clearer in the revised manuscript. (p. 5)

The paragraph now reads as:

“The original realization factors framework lacks clear operationalization because it only offers broad definitions of each factor and a few examples of organizational strategies. In the following sections, based on our scoping review, we detail specific organizational strategies and outcomes under each realization factor to help unpack the connection between hospital goals (MVV statements, strategic plans, and other representations of goals) and PE practices (e.g., aligning goals across organizational documents, elaborating on strategies that align processes with hospital PE goals, and describing ways to provide support and resources to employees to integrate PE into their work).”

**Barriers / Strategies: I feel that signposting at the start of each realization factor heading, that there was, for example, one major barrier elucidated from the review and five strategies (then numbering each e.g. Strategy 1, strategy 2 etc…) will create additional clarity to the manuscript. As an example, I would therefore suggest bringing forward the paragraph “In summary on page 10…. to become the preamble to the section on Barrier and Strategies on page 9 rather than the conclusion. Same for the motivation and empowerment sections. I like the approach used just make it explicit and upfront for the reader.**

Thank you for this comment. We agree that there is a need for better clarity in the signposting of each subsection of the findings section. We have addressed this by elaborating our signposting by clarifying the specific barriers and strategies discussed in each subsection. We also have a summary table at the beginning of the synthesis section that describe each barrier and strategy (pgs. 7-12).
